# The Experience of Self-conscious Emotions in Inflammatory Bowel Disease: A Thematic Analysis

**DOI:** 10.1007/s10880-021-09778-0

**Published:** 2021-12-23

**Authors:** Noelle Robertson, Sarah Gunn, Rebecca Piper

**Affiliations:** 1 Neuroscience, Psychology and Behaviour, University of Leicester, University Road, Leicester, LE1 7RH UK; 2 Clinical Psychology, University of Leicester, Centre for Medicine, Lancaster Road, Leicester, LE1 7HA UK

**Keywords:** Self-conscious emotions, Shame, Inflammatory bowel disease, Thematic analysis, Qualitative research

## Abstract

Few studies have investigated emotional experiences in people living with inflammatory bowel disease (IBD). However, self-conscious emotions, including embarrassment and shame, are indicated as a key factor in delayed help-seeking for bowel symptoms, which can result in poorer health outcomes. This study aimed to explore experiences of self-conscious emotions among people with IBD. Fifteen participants were recruited from outpatient IBD clinics and patient groups, and engaged in semi-structured interviews about their experiences of IBD-related self-consciousness. Data were analysed using thematic analysis following an inductive, semantic approach and conducted from a critical realist position. The analysis generated two themes, each with three sub-themes, which captured self-conscious emotions in relation to experiences which threatened participants’ preferred identities. The first theme, ‘Lack of control’ encapsulated participants’ distress relating to fundamental alteration in self-perception, and their attempts to mitigate this. The second, ‘Lack of understanding’ captured distress associated with awareness of being unfairly judged by other people. Clinical implications are identified, including consideration of therapeutic approaches which target self-conscious emotions such as shame, and continued societal efforts to educate others about invisible disabilities such as IBD. Experiences which threatened participants’ identities were implicated in the generation of self-conscious emotions; these should be considered in work with clients with IBD. Future research should target further investigation of these constructs.

## Introduction

Inflammatory bowel disease (IBD) describes chronic gastrointestinal conditions, most commonly ulcerative colitis (UC) and Crohn’s disease (CD). Both can cause diarrhoea, pain, weight loss and fatigue, linked to inflammation and ulceration within sections of the digestive system (Crohn’s & Colitis UK [CCUK], 2014), as well as extra-intestinal manifestations such as skin rashes and joint or eye inflammation (CCUK, 2014). IBD is estimated to affect up to 1% of the UK population, with high lifetime costs, and is most commonly diagnosed in late teens and early adulthood (Lichtenstein et al., 2020).

As for many other chronic conditions, growing evidence recognises both biological (immunological and genetic) and psychological predispositions in IBD (Bitton et al., 2008; Mackner et al., 2011; Mawdsley & Rampton, 2005). Of particular importance is the recognition that the relationship between the brain and gut microbiome is bidirectional, with psychological difficulties (symptoms of anxiety and depression) both affecting and affected by IBD symptoms (Gracie et al., 2018). New-onset gastrointestinal symptoms are an identified trigger for psychological disorder, and can develop themselves in response to psychological distress (Gracie et al., 2019). Therefore, a full understanding of IBD must comprise a holistic overview of these factors. In this study, we explore the role of self-conscious emotions in mental wellbeing in people with IBD, which have been little-studied and yet may play an important contributing role in illness-related distress.

### Psychological Factors in IBD

Psychological difficulties are common in IBD. People with IBD report consistently higher anxiety and depression than healthy controls, and mood disorders are associated with higher disease severity, raised risk of relapse and greater likelihood of surgery (Graff & Dudley-Brown, 2013; Mikocka-Walus et al., 2016). People with IBD also report poorer quality of life, particularly when IBD symptoms are more active (Dibley et al., 2018; Robertson et al., 2019; Sajadinejad et al., 2012; van de Star & Banan, 2015). Poor mental wellbeing is clearly complex in IBD, and developing an understanding of the role of self-conscious emotions in IBD is warranted.

Self-conscious emotions are an important facet of emotional experience in chronic illness, with growing awareness of the role of shame in adjustment to chronic conditions such as diabetes, pain, HIV and chronic obstructive pulmonary disease (COPD) (Archer, 2014; Batchelder et al., 2018; Browne et al., 2013; Harrison et al., 2016; Werner et al., 2004). High levels of negative self-conscious emotion have been associated in some cases with riskier health behaviours, for example in people with HIV (Batchelder et al., 2018). Self-conscious emotions are also potentially key in IBD given the social threats incurred through urgency and the possibility of faecal incontinence, which can provoke shame and embarrassment and adversely affect self-identity (Cooper et al., 2010; Hall et al., 2005; Keeton et al., 2015; Kemp et al., 2012; McCormick et al. 2012; Schneider & Fletcher, 2008). To date, there has been little qualitative exploration of the influence on self-conscious emotions on wellbeing in IBD; however, such exploration could enrich our clinical understanding of the lived experience of IBD and its management.

Self-conscious emotions involve cognitive appraisal of one’s behaviour in relation to internalised ideals (Tracy & Robins, 2007). In social self-preservation theory (Gruenewald et al., 2007) these emotions are argued to arise when individuals experience social threat, e.g. after violating a social rule. Despite increasing research attention, self-conscious emotions are often poorly conceptualised. We use this umbrella term here to describe emotions which heighten one’s self-awareness, serving to protect social status and promote positive social relationships.

Five specific self-conscious emotions have been identified as potentially relevant to IBD; (1) embarrassment, a fleeting experience of awkwardness and regret over unexpected incidents, which motivates generosity and helping behaviours (Miller, 2007); (2) shame, an internal attribution of oneself as “bad” and “inferior” to others when perceived as unattractive, inadequate or flawed (Gilbert, 1997, 1999, 2002); (3) humiliation, experienced when placed in a powerless state by another, which is externally attributed to a ‘bad’ and unjust other (Gilbert, 1997, 1999); (4) self-disgust, a more extreme place on the shame continuum (Simpson et al., 2010), associated with appraisal of oneself as repulsive to others (Powell et al., 2015), and particularly salient for those with bowel disorders who fear being seen as “contaminated” due to their symptoms (Ekströmer, 2002), and; (5) guilt, experienced when one’s behaviour is believed to have had negative consequences, which motivates apologetic and reparative actions (Tangney, 1996).

Self-conscious emotions are noted as important in the circumscribed qualitative research that has explored experience of IBD in specific groups, notably young adults (Daniel, 2001), individuals post-surgery (Allison et al., 2013), and those self-managing the condition (Cooper et al., 2010; Hall et al., 2005; McCormick et al., 2012; Schneider & Fletcher, 2008). Findings allude to embarrassment and distress, which delay treatment-seeking and ultimately harm health outcomes due to later diagnosis/intervention (BMJ, 2012; Schoepfer et al., 2013). While Kemp et al. (2012)’s metasynthesis of research into IBD experience identified important tensions between drives for social engagement and social isolation due to fearing disease-related incontinence, the authors noted that the existing qualitative literature did not adequately capture the emotional repercussions of living with IBD. This gap in the literature has not yet been addressed. Accordingly, further qualitative exploration of self-conscious emotions in IBD is warranted, particularly given the potentially harmful effects of delaying treatment-seeking.

### Rationale for Present Study

Prior research has largely been quantitative, and this has identified a potentially important role for self-conscious emotions in IBD. However, qualitative exploration of the lived experience of IBD in relation to self-conscious emotions is essential to understand the repercussions and contexts of these negative experiences. Developing such an understanding may be key for conceptualising emotional difficulties associated with IBD, and their impact on individuals with the condition. This will be highly applicable to therapeutic work with such individuals. Accordingly, in this study, we explore the presence and impact of self-conscious emotions for people with IBD.

### Study Aims

1. To explore the experience of self-conscious emotions in people with IBD.
2. To understand the psychological and social impact of self-conscious emotions on individual’s lives, relating to IBD symptoms and lived experience.

## Method

### Design Overview

This qualitative interview study utilised thematic analysis (Braun & Clarke, 2006) to explore participants’ experiences of self-conscious emotions related to IBD while enabling flexibility in constructing bespoke analyses. An inductive, semantic thematic analysis was therefore designed, in accordance with the research questions and our epistemological position. Semi-structured individual interviews enabled detailed accounts to be shared by participants, with scope for more exploration of areas of particular interest, while enabling privacy for discussions about sensitive health issues and associated self-conscious experiences.

We adopted a critical realist position (Willig, 1999), recognising ourselves as active participants in the research process, mindful of our own experiences and values in relation to clients and family members with IBD diagnoses when shaping the study and interpreting results.

### Participants and Sampling

Participant eligibility criteria were deliberately broad, in order to understand experiences across heterogenous people living with IBD. The criteria comprised:

- Holding an IBD diagnosis (Crohn’s disease or ulcerative colitis) from a medical provider for 12 months or longer, to allow a period of adjustment to living with the condition.
- Aged 18 years or older.
- Possessing sufficient English fluency to engage in the semi-structured interview.

Participants who received their diagnosis of IBD prior to leaving secondary education were not eligible, given that school experiences may affect self-conscious emotions differently to those in adulthood (Day et al., 2012). Participants with comorbid chronic illnesses diagnosed within the last year were also excluded, as it was felt that coping with a new diagnosis could confound the findings relevant to IBD-related distress. However, given that many people with IBD have comorbidities, some of which are also potentially related to the condition and the systemic inflammation associated with it (Argollo et al., 2019), excluding those with comorbid conditions entirely could create a biased sample which did not realistically reflect life with IBD for many individuals. Consequently, those with comorbid diagnoses made prior to the year before inclusion were still eligible to participate. These criteria were made clear in the recruitment media.

Fifteen participants contributed to the study (for characteristics, see Table 1, with pseudonymised participants and limited demographic information to maintain anonymity). This number was considered to be appropriate and consonant with common practice in research studies seeking to identify patterns across qualitative data (in which a sample size of 15–30 is typical) (Braun & Clarke, 2013). Participants were recruited via outpatient clinics and patient groups from gastroenterology departments in two acute NHS Trusts in the UK Midlands. A recruitment poster was displayed in communal areas of IBD clinics at each centre, with specialist IBD nurses providing brief information leaflets to potential participants meeting inclusion criteria. CCUK patient groups associated with participating Trusts were similarly informed and the poster shared on local CCUK social media sites. Nine participants were recruited through hospital clinics, the remainder through social media, and a minority (three) were not connected to patient support networks. All participants recruited completed the study, with no need to exclude post-recruitment as eligibility criteria were made clear in the recruitment materials and this successfully pre-filtered individuals prior to their making contact. After the 15 recruited individuals had completed, it was judged that data saturation had been reached. Participants’ age ranged from 25 to 75 years, with duration since diagnosis of IBD ranging from 4 to 20 years. Three participants reported that they had undergone ostomy surgery.

**Table 1:** Participant demographic information

Demographic characteristic	Number of participants
Male/female	4/11
Age in years (range)	25–75
*Ethnicity*	
White British	13
Ethnic minority	2
*Relationship status*	
In a relationship	9
Not in a relationship	6
*Diagnosis*	
Crohn’s disease	9
Ulcerative colitis	4
Mixed	2
Years since diagnosis (range)	4–20

### Data Collection

Interviews took place from November 2016 to March 2017. Participant information sheets were provided to those expressing interest who could then opt-in and attend an interview, at which they provided informed consent. Thirteen interviews took place at participants’ homes, and two in neutral locations at participants’ request. All interviews were audio-recorded and duration ranged from approximately 40–130 min.

Participants were invited to share experiences of self-conscious emotions linked to their condition. A pre-developed interview topic guide with prompts to assist the elaboration of answers was used to ensure areas of interest were given adequate consideration. This guide was developed through consideration of existing literature, in consultation with an IBD specialist nurse and clinical psychologist working with people with IBD. Audio-recorded data were saved on an encrypted memory device and transcribed verbatim following each interview, with pseudonyms allocated and identifiable information removed.

### Analysis

Having checked transcript accuracy against audio-recordings and considering contextual information, coding and organisation of the data was conducted using QSR NVivo 10 software, and analysis undertaken via thematic analysis as articulated by Braun and Clarke (2006). Initial familiarisation was obtained through each transcript being rigorously read by one author who generated an initial set of codes. Codes revealing similar meaning were grouped, with these clusters achieving internal coherence via further review and revision. Codes considered less salient to the research questions (e.g. relating to medication) were excluded from the present analysis. Discussion between authors aided reflexivity (already prioritised via keeping of a reflective journal (Tracy, 2010; Yardley, 2000) and regular research ream meetings) and theme clarity, facilitating coherent theme organisation and use of suitable illustrative quotes. These regular meetings also enabled monitoring of thematic saturation in participant data, and developing consensus around emerging themes. Final themes were examined for accuracy by all authors to ensure that content captured the transcript data. To enhance readability, hesitations and repetition have been removed from extracts used (Sandelowski, 1994).

### Ethical Considerations

Ethical approval was granted from the Health Research Authority in September 2016 (reference: 16/WM/0290) and the Research and Development departments of the supporting NHS Trusts granted approval to advertise the study via their premises. Written informed consent was gained from each participant and confidentiality was maintained throughout. Study information was shared in both oral and written format with potential participants, to ensure comprehension. Information about available support services was shared with all participants post-interview.

## Results

**Fig. 1:**
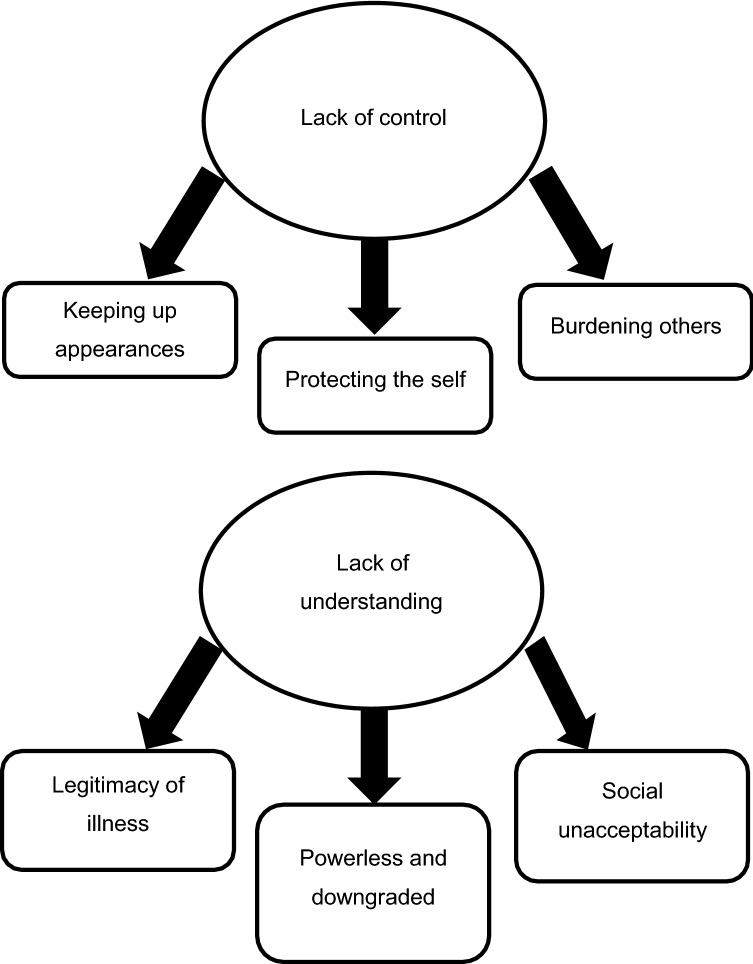
Map of generated themes

Although many participants reported that living with IBD had become easier over time, all spoke about times when IBD had caused significant difficulties and intense distress. Remission was regularly cited as reducing condition-related distress, as was familiarity with IBD and management of symptoms. Regarding sources of self-conscious emotion, all participants described experiences of their identity feeling threatened. These identity threats were articulated via thematic analysis as two major themes: perceptions of their own behaviour as somehow ‘wrong’ due to disease processes outside their control (“*lack of control*”), and perceptions that others believed their behaviour to be ‘wrong’ due to ignorance of the disease and its effects (“*lack of understanding*”) (see Fig. 1). Each theme was supported by three sub-themes, which provided additional detail.

Throughout the analysis, we used the definitions for self-conscious emotions outlined in the introduction as our frame of reference when interpreting participants’ accounts. At times, therefore, the analytic narrative may use different terms to participants’ reports. However, we aimed to remain congruent with the apparent intended meaning.

### Theme 1: Lack of Control

This theme captures participants’ experiences of their preferred identity being threatened by lack of control, caused by the disease or its effects becoming visible. Participants endeavoured to preserve a disease-free identity and regain control. Some reported feeling fear and anxiety that this identity might fail to be maintained, shame when this occurred, and guilt when disease effects created difficulties for others. Three sub-themes were generated.

#### Subtheme 1: Keeping Up Appearances

All participants emphasised wanting to retain their usual persona, rather than being perceived as different. However, the nature of IBD, involving odours, urgency and systemic effects such as fatigue, threatened these preferred identities. Janet described the consequences of faecal incontinence:

When I do have an accident I'm just devastated, and I don't think I'll ever get used to that, because it is a loss of control isn't it? It's not just a loss of control of your bowels but just…you feel like you're losing control of yourself.

Janet’s words encapsulate the distress generated by lost control of bodily function, and how this threatened her self-identity. Erica described similar negative emotions:

Embarrassment at having to go and vomit and embarrassment at my abdomen being so noisy, and having wind, and some shame with that as well, cos […] it's not the way you want to be perceived, it's not the way you want to live.

Other participants echoed this fear of others’ negative perceptions: “Cos it smells… it might be noisy […] the worst thing is thinking—especially if there’s a queue and someone’s got to go in there afterwards—and they know it’s you” (Jennifer); “…embarrassed that people might come in…and see my nappy round my ankles […] it's not something to be proud of” (Ian). Potentially being seen as ‘different’ and judged negatively was shameful.

Shame-related distress was prominent when disease sequelae intruded on intimate relationships. Katie reluctantly sought IBD-related help from her partner, but feared her relationship could be threatened: “I wouldn't look at him, and he was just like, turn around so I can actually help you”. Katie averted her body and avoided eye contact, behavioural manifestations of shame (Keltner & Harker, 1998) which suggest she felt her attractiveness was jeopardised.

Uncontrolled bodily functions were a clear source of self-conscious emotion. However, fatigue and changes in physical appearance also threatened participants’ identities. Ben described the impact of weight loss after a hospital stay: “it really hit home when I was standing in front of a long mirror at home”. Similarly, David was self-conscious after stoma surgery: “even though you could only see a tiny bit of it…that tiny….kind of…5 mm at the top that…come above my shorts…in my head felt like…it was the size of a boulder”. Physical changes challenged the participants’ constructions of themselves, by demonstrating their ill-health to others. These examples illuminate the sense of being different caused by IBD, and the consequent threat to participants’ previously healthy identities.

Many also avoided telling others they felt unwell, for fear of appearing abnormal. Erica described:

Knowing myself I couldn't do things, but not wanting others to be telling me I couldn't, or acknowledging to other people, apart from those very close to me that I couldn’t do it, trying to keep up the appearance of…being able to do things.

This sentiment was expressed by several participants. Donna stated: “I wouldn't want to be defined by it […] It's an illness rather than me”. This illustrates the crucial perceived importance of retaining and portraying an identity separate from IBD.

#### Subtheme 2: Protecting the Self

Participants reported several strategies to prevent others becoming aware of the illness, and thereby preserve dignity and mitigate shame: these were hiding, avoiding, planning, overcompensating, use of humour or deflection, and attempting to explain or justify their behaviour. Participants avoided situations or discussions about their health, to preserve their healthy-seeming persona. Sally described “I just wanted to be at home because it is so embarrassing”; Ben “did spend a lot of time at home”, while Jennifer said “you learn to not show it”. Similarly, Donna explained that “I always say, "I'm fine thanks", always, no matter how rubbish I am”, and James said “I wasn’t gonna begin to tell them [friends] or speak to them about it”.

Several participants adopted secretive behaviours to hide their illness. Erica described visiting a different area in her work to use toilets anonymously:

I'd go to [next door] and be sick in their toilets and come back again, and nobody really questioned it. [Interviewer: Why is it important to do that?] …to hide it, because […] I didn’t want people to know I was so bad. Because I was in charge, and so therefore…you have to…erm…be reliable.

Similarly, Melissa described attempts to disguise frequent trips to the bathroom during work time:

Sometimes, I come back [to my desk] with a piece of paper or a brochure, so it looks like I've been somewhere [laughing] […] Or take a notepad with me, and then we've got a table in the toilet area, I can just leave it there…walk back with my notepad! Think I'm quite clever when I do that! [Laughing] [Interviewer: Why is it important to do that?] Oh, just so it's less obvious. Looks like I'm at work, that I'm working and…yeah. Just try and…conceal it.

These elaborate behaviours enabled protection of highly valued professional identities.

Planning ahead was unanimously identified as a strategy to prevent IBD from interfering with life. Janet would “not eat the night before” when she had plans the next day, which was described by several participants. Ben would “keep [his] car well stocked of…you know…diarrhoea tablets and…paracetamol and everything”. Ian said: “I've now got a toilet in the car, so I'm out in the car, I'm not gonna get caught out”, and Gemma explained that “I’ve learnt to put toilet roll down there to stop the…but you know, you do get your noise sometimes when you’re being sick…”. Several participants also changed employment, or opted for self-employment to allow greater control of their working environment.

Maintaining appearance offered a further self-protective strategy. Katie was one of several participants who described taking particular care of her hair and clothing, to distract from signs of disease and display a self with which she was comfortable: “Because there was something wrong inside, I didn't necessarily want there to be anything wrong outside”. Humour was also deployed to deflect attention from symptoms; for example, Jennifer described feeling conscious of the side-effects of medication: “I always got in there first […] I think that’s what I do to kind of protect, I’m like, oh my God, excuse my horrible, round, fat, hairy face”. Similarly, Alice shared that she used body-spray to mask smells caused by IBD, explaining: “I made it into a joke for me to feel better”. By acknowledging their disease before others could, participants pre-empted potential censure for any perceived behavioural aberration.

#### Subtheme 3: Burdening Others

When attempts to hide illness failed, participants acknowledged feelings of responsibility for others. Joan described instances of perceiving herself as a burden to others, notably when needing swift access to toilets or support from family with managing her stoma: “I do feel so humiliated by it all. I feel like…rubbish – a complete waste of time”. She elaborated:

I started to think, “this is my fault”. And I’m spoiling people's lives and making life difficult for them. […] No one ever, ever suggested it—I was never shown that but it was what I started to think. I’m a nuisance really.

Joan was discomfited, expressing the sense she had unwilling broken an internal standard of not troubling others. For others such as Gemma, guilty feelings were also associated with exposing others to unpleasant bodily fluids or odours: “I wouldn’t want a mum to go in there with a new baby and smell, you know, what I’ve been through”. The knowledge that she might adversely affect others provoked self-consciousness.

Participants were similarly hesitant to cause relatives worry regarding their wellbeing. Some attempted to protect close others and reduce their own guilt by limiting what they shared:

I don’t really want to think there’s someone worrying about me all the time […] [mum]’ll say, “how’s your stomach?” and I’m like “yeah, yeah it’s fine” and she’s like, “you wouldn’t tell me if it wasn’t would you?” I was like, “No!” (Jennifer)

Similarly, James noted that “I don’t really want my parents to worry […] so I probably don’t really say that much to them at all.

Participants also experienced guilt around restrictions on family life. Gemma stated: “you feel guilty cos you can’t do stuff as a family together” when explaining the impact of IBD-related joint pain. Ben also acknowledged the restrictive impact of fatigue: “when I'm lying on the sofa…I feel that I should be playing with the kids”. Other participants acknowledged more sustained guilt regarding opportunities missed: “It's the guilt that they've got older now so…you can't get it back […] that's the bit that's constantly…gnawing away” (David); “I felt guilty that my illness was putting…this pressure and worry on [children], when they…should have been able to…enjoy their teenage years, like all their friends were” (Erica). These experiences again demonstrate the discomfort emerging from the impact of participants’ IBD on others.

This also applied in the workplace. Participants described their IBD undermining work roles and capacity, and putting pressure on colleagues: Gemma said: “Once I do need the toilet, I can’t hold it, and I’m thinking are they going to be short-staffed, and the smell…”; Katie described “I'd need to have time off and I'd feel then guilty about having time off”; and Melissa stated “Just the thought of myself…letting myself down, letting the company down…”.

Within intimate relationships, participants described physical and emotional impacts of self-consciousness, as described by Erica:

I couldn't stand anybody to put their arm over me […] I think like anybody…who's got Crohn's or colitis and you're in pain or whatever, it affects….it affects your sex life because you, you feel, I think your partner is worried about…you not being well enough, and you're worried about…for me it was…it was passing wind or….it being painful, you know, if he'd touch me or put his arm round me or whatever, you know, so yes, it does, it does affect your personal life…

Participants described constraints on relationships and physical contact as invoking guilt: “He shouldn't have to deal with someone being sick” (Katie); “I feel really sorry for [partner] at the minute cos I don't even wanna give him a cuddle, you know?” (Erica). Indeed, some participants chose not to seek intimate relationships at all. Alice described: “How can you sit in a man’s company, when you trump—to put it politely—probably worse than they do, and there’s nothing you can do?”.

I don't think anybody would want to live…with…with somebody with an illness either…it's not fair on somebody else is it? […] I don’t think I’d like to share a bed with anybody anymore…in-in case I have…ever had an accident, cos that would be awful. (Donna)

In addition to desiring to protect others from the disease, these quotes indicate that self-perception can be affected; this might be understood in terms of shame, with IBD threatening one’s perceived attractiveness.

### Theme 2: Lack of Understanding

This second theme encompasses participants’ identities being threatened by others’ lack of understanding of IBD and its effects. Emotions evidenced in this theme include humiliation, anger, indignation and resentment at perceived unfair treatment. Again, this theme comprises three sub-themes.

#### Subtheme 1: Legitimacy of Illness

All participants had experienced others questioning the validity of their illness. Explicit and implicit messages were received implying malingering, questioning the legitimacy of symptoms, or dismissing the condition as something less serious (e.g. irritable bowel syndrome). James explained that he needed to fight to be taken seriously by doctors: “being treated as if you’re stupid, or being treated as if you’re a hypochondriac”, an experience common to several participants which provoked indignation. This also led participants to question themselves: “…you don't want to do anything about it because you think, well, is it in my mind?” (Sally). Experiences of being dismissed often prompted avoidance of further help-seeking, risking health deterioration.

Relatives and friends could also be unsympathetic. Erica shared her frustration:

If they do believe you they think it's just…you know, you get people…saying, "oh well I've got IBS sometimes, you know, and I get some cramps", and I think, you have no idea. And I say "oh yes, it's hard isn't it", you know. But they have absolutely no idea, when you're on your hands and knees for…6 h…breathing through the pains like you're in labour […] but really, it's only that…these people don't know. They think that their…little twinges…or having to go to the loo twice a day is the equivalent to somebody sitting for four hours with bloody diarrhoea.

Laypersons’ lack of understanding of IBD may consequently create conflict for participants who want acknowledgement of the seriousness of their condition.

Participants also shared experiences of being publicly questioned about their need to use disabled toilets, which was experienced as intensely unfair. Ian explained that problems with urgency meant he commonly experienced such judgement:

People are looking and thinking… “nowt wrong with you”, you know. I had one a couple of weeks back, a lady waiting in a wheelchair, and I come out…and she's like, "what's up with you?" […] When I'd come out of a disabled toilet, I'd always try and listen if I could hear anybody out there, and always try and sneak out so no one sees me coming out, because I am always a bit…ashamed of using one.

Such challenges were associated with highly aversive public humiliation; Ian would even simulate visible physical impairment at times to justify his use of disabled facilities. By contrast, other participants would respond more indignantly:

I say, "it's none of your business!" And they say, "well why do you need to use it?". "Because I need to use a disabled toilet." "But you don't look disabled?" So, the one time I got really angry and I said to this woman, I pulled down the top of my trousers, and I said, "well I've got an ostomy bag, do you wanna have a look?” (Katie)

Participants felt that legitimacy could often be achieved only by demonstrating some overt aspect of IBD or directly informing the accuser of their condition. Katie further explained that although she resented showing others her ostomy, she felt making her illness visible afforded greater authority and made her behaviour ‘defensible’.

#### Subtheme 2: Powerless and Downgraded

This subtheme captures feeling judged by others, which was evidenced in situations where participants received unjustified comments, or when their condition was unwillingly exposed. All participants described experiences of feeling patronised, infantilised or an object of contempt due to their IBD.

Participants often reported being placed in a ‘sick role’ by others, related to faecal incontinence and/or vomiting. Erica described: “it demeans you a bit. […] you feel like you've either become a child or an elderly person”. Katie echoed this sentiment regarding how her partner monitored her: “sometimes that gets very motherly […] that’s what my mum does”. Joan described her intense distress as others judged her for causing an unpleasant smell: “I felt a freak actually…I felt—you know like a small child that didn’t know what to do with […] poo and stuff. It was horrendous”. Such responses from others could engender humiliation and self-disgust. Janet reported such feelings when being publicly alerted to her own faecal incontinence by a stranger:

I was just mortified that she'd sort of mentioned it even if…she did it […] in a kind way…I don’t know that I would be so brave […] to go up to somebody and say “oh I think you’ve had a…”. I think I would just sort of…ignore it really. Erm…I know that's what I guess I want people to do, but…sometimes it's […] visible that you've…you know, you have had an accident, so it's not…you know, it's not as if you can hide it, is it? But yeah, I'm just mortified.

Having one’s bodily functions made obvious through strangers’ comments was experienced as socially devaluing.

Many participants held ‘can’t wait’ cards, designed by CCUK to gain urgent access to toilet facilities. However, using these cards forced participants to reveal their condition and this was often avoided, as Ian acknowledged: “to get over my embarrassment enough to use it…I would be at the point where I'm literally about to poo myself”. David also reported that a customer aware of his IBD diagnosis refused to be served by him: “just the name itself…scared her”. Participants’ descriptions of their need to request permission (evoking toilet break requests at school) and being treated as ‘contagious’ was experienced as belittling.

Unhelpful comments were also experienced in the workplace, as Katie explained: “I didn't quite believe that some people would say things like that”; and Melissa described her manager’s response to a recent flare: “don't rush back, cos I don't want you to be a distraction [to other employees]”. Both explained how these experiences contributed to longer absences and reduced self-confidence.

#### Subtheme 3: Social Unacceptability

Participants shared an implicit understanding that discussing their IBD was taboo, as Melissa identified when recalling embarrassment in a return-to-work interview after a hospital admission:

I was like well embarrassed, he was like, "err, so what was wrong?". I was like, "I…I don’t…I can't", oh, yeah, just embarrassed…to talk about things like that, I know it's like really an old school taboo subject thing that people should talk about but…I dunno, I don't talk about going to the bathroom with people.

Like most participants, Melissa found discussion of bowel habits impolite and uncomfortable. Breaching this tacit social rule created a sense of rupture, as Janet revealed from her support group for people with chronic illnesses:

I was the only one with inflammatory bowel disease and every time it got to talking about that…the whole group just shut down. […] It was quite acceptable for people with MS [multiple sclerosis] to talk about being incontinent of urine, and they could deal with that and they could talk about that. But when I broached, you know, losing the control of bowels, it was just like…even the trainer couldn't bring it back. […] It made me feel a bit like a leper! I felt like I was…socially unacceptable really.

Even in supportive environments, talking about taboo subjects revealed that others seemed ill-equipped to respond. Joan noted a friend’s disgust response to disclosure about her condition: “I couldn’t help but notice her face, this sort of pulling her face thing as much as like, oh how revolting”, which triggered shame and humiliation.

Perhaps in reaction, participants described becoming highly attuned to others’ responses during interactions. Katie described: “a sense when I'm talking to somebody that we needed to…erm…change the topic of conversation by the massive pause after I've said something and then they talk about something else quite quickly”. Participants identified the necessity to calibrate interactions, and spoke of frustration at needing to censor an important part of their lives. Involvement in support groups provided a positive outlet for this frustration, enabling participants to advocate for and support people with IBD. David explained: “why I do all these charity things […] I can't see why anyone else should have to go through what I did, so…you gotta do summat about it”. Several participants expressed similar sentiments, sharing a desire to improve things for others with IBD and to address social isolation and self-consciousness.

## Discussion

We explicitly sought to understand self-conscious emotions experienced by people with IBD, and their impact on participants’ lives. Rigorous thematic analysis elicited two broad themes encompassing participants’ experiences: ‘lack of control’, which focussed on participants’ perceived divergence from a ‘normal’ ideal self as a consequence of IBD, and ‘lack of understanding’, which focussed on other’s crass behaviour towards participants, attributed to poor knowledge of the disease.

### Findings in Relation to Previous Literature

Participants’ experiences resonate with Kemp et al.’s (2012) metasynthesis exploring IBD experience, which highlighted similar themes including fears of incontinence, desire for control, and difficulties arising from the invisible nature of IBD. The present study extends these findings by considering the emotional impact of these experiences.

Key to narratives was that disease experiences appeared to threaten participants’ social standing. Threats emerged through IBD-related changes to bodily functions and physical appearance, and others’ interactions with participants which communicated contamination or inferiority. ‘Lack of control’ was associated with participants’ embarrassment and shame in their inability to control their bodies, and others witnessing these struggles. ‘Lack of understanding’ encapsulated participants’ appraisals that they were treated unfairly and humiliated, and a wish to assert themselves on their own behalf and for others with the disease. Such self-conscious emotions, especially around shame, humiliation and lack of compassion, reflect those identified in other chronic conditions including diabetes, long-term pain, HIV and COPD—and similar concerns about others’ judgement and a need for concealment of conditions in relation to these emotions (Archer, 2014; Batchelder et al., 2018; Browne et al., 2013; Harrison et al., 2016; Werner et al., 2004). That emotional responses for those living with IBD resonate with those reported in diabetes, HIV and COPD is interesting; IBD is not blameworthy, in the way risk for these other conditions may be seen to imply personal culpability through lifestyle choices. Yet the similarity in extent and focus of self-conscious emotions for those with IBD suggests the potency of a disease to invoke taboos from childhood regarding mastery of basic bodily functions alongside profound disgust sensitivities, even when the disease implied no personal responsibility for cause.

The distinctions between themes can be understood in terms of Gilbert’s (1997, 1999) conceptualisation of shame and humiliation. While experiencing shame, individuals perceive themselves as deserving of others’ poor opinion, while in humiliation, individuals feel undeserving of negative opinion. Where participants perceived others as unfairly doubting the legitimacy of their illness or their use of disabled facilities, they experienced anger and humiliation. However, when participants felt responsible for controlling IBD manifestations and ‘failing’, they experienced shame, likely due to a breach of social norms in which adults are expected to have full control of their bodily functions. People with IBD may feel that visible evidence of such bodily ‘incompetence’ leads them to be stigmatised by others, and concealment affords emotional control (Dibley et al., 2018). Stigma is known to contribute heavily to health-related quality of life in IBD, and its influence is clearly important (Taft et al., 2009), considered to adversely impact psychological wellbeing through discrimination and compromised social status (Taft & Keefer, 2016).

Shame and humiliation can be conceived with regard to the evolutionary ‘fight or flight’ response (Gilbert, 2001, 2002). Shame, concerned with protecting one’s social attractiveness, encompasses ‘flight’ responses of escape, avoidance, or appeasing others; this was echoed in participants’ behaviours, notably avoiding social situations and using self-deprecating humour. In contrast, humiliation, experienced when one is unwillingly and undeservedly made powerless, encompasses ‘fight’ responses in which individuals feel obliged to mount a counter-attack to maintain social standing. This was resonant with participant behaviours such as aggressively demonstrating their illness and overtly supporting others with IBD.

Participants’ accounts indicated switches between hiding and asserting their IBD, which can be understood through the ‘shifting perspectives’ model of chronic illness (Paterson, 2001). This suggests that ‘illness’ and ‘wellness’ shift fore and aft depending on perception of symptom burden, and on identification with or distance from an ‘unwell’ identity. During periods where participants were oriented to ‘wellness’, they might be more likely to hide their illness, and when focus was on ‘illness’, they may have been more open. Such shifting parallels the episodic nature of IBD. Dibley et al. (2018) also describe a shifting pattern of sensed stigma, dependent on the relational context of negative experiences such as bowel accidents—notably, the authors describe “kinship stigma”, in which negative attitudes from relatives and close others can be extremely distressing. This concurs with the expressed experience of the participants in this study.

Participants’ propensity to experience shame or humiliation may depend on self-perception prior to becoming unwell, potentially shaped by early experiences and attachment style (Martins et al., 2016). Experiences of rejection and criticism with a primary caregiver may promote an internalised view of oneself as ‘bad’, unworthy and deserving of poor opinion from others (i.e. more inclined to shame), whereas non-shaming developmental experiences would facilitate feelings of being worthy and acceptable to others (potentially inclining more to humiliation) (Schore, 1998).

That participants experienced guilt when their IBD negatively affected others (i.e. when family life was constrained or colleagues exposed to disease effects) is understandable, given humans prioritise maintaining social relationships (Tangney, 1996). Our need to belong may explain why those most self-conscious about IBD avoid social situations, stop seeking new relationships, and make reparations where IBD has encroached on others’ lives. Such avoidance may magnify isolation, affect mood and confirm negative beliefs of themselves and social desirability, which in turn may damage mental health (Scheff, 2012).

### Clinical Implications

Access to specialist psychological support is increasingly recognised as key for successful management of IBD (IBD Standards, 2019). Given current understandings of the bidirectional interaction between wellbeing of the brain and gut (Gracie et al., 2018, 2019), this appears even more crucial. Our findings suggest that time and opportunity to understand the emotional repercussions of IBD should be explored in routine consultations, and where appropriate, referral to psychological therapists enacted to offer thorough assessment. Therapeutic work with psychological practitioners may also be a useful adjunct alongside routine consultations and follow-up in gastrointestinal clinics, to help staff elicit patients’ self-conscious concerns and consider their relevance to management of IBD.

Given the size of our study sample, we cannot assume that all individuals with IBD will experience potentially disabling self-conscious emotions. However we believe our findings do highlight the importance of self-conscious emotions for those living with IBD. Incorporating routine assessment to discern the risk and focus of such emotions (such as internal versus external shame, body shame, or humiliation) through robust validated assessment tools can both identify extent of clinical need and suggest therapeutic focus. Such assessment seems a key adjunct to the current delivery of CBT, which has been deployed to manage other psychosocial sequelae of IBD such as fatigue and poor quality of life, with good initial results (Artom et al., 2019; Gracie et al., 2017). However, CBT has shown more equivocal impacts on disease activity, coping, and mental wellbeing (Mikocka-Walus et al., 2017).

Where self-conscious emotions such as shame and humiliation are prominent, third-wave therapies may be indicated to support emotional regulation and mitigate the impact of isolation emergent from perceived challenges to social status (as described above). Appropriate avenues might include nuanced therapies such as compassion-focussed therapy, which can mitigate shame (Gilbert, 2009), or acceptance and commitment therapy, which aims to increase psychological flexibility and acceptance of difficulty (Graham et al., 2016; Gutierrez & Hagedorn, 2013; Luoma et al., 2012). The targeting of self-conscious emotions alongside adapted CBT interventions may further enhance clinical outcomes—however, this requires further research.

In a more systemic sense, strong support networks appear to benefit emotional wellbeing in IBD, indicating the importance of patient support groups and encouraging individuals to foster connections within their personal networks. Our findings also concur with previous work showing that such support reduces the impact of stigma in IBD (Dibley et al., 2018).

Promotion of bowel disease awareness remains a key task for organisations such as CCUK, who produce the ‘can’t wait’ card, advocate for toilet signage changes to acknowledge hidden disabilities, and campaign with high-profile figures with IBD to encourage awareness. However, participants described numerous examples of others’ lack of awareness provoking distressing self-consciousness, notably gate-keeping of toilet access in shops and questioning use of accessible toilet facilities, which suggest further advocacy is needed.

### Strengths and Limitations

This is the first study to explicitly investigate experiences of self-conscious emotions in people with IBD. In doing so, it offers novel insights regarding experiences of shame and humiliation emerging from social threats to identity, and argues for enhanced awareness, assessment and access to tailored therapies. However, sample size was slightly smaller than that typically advocated for thematic analysis, although data saturation is achievable with as few as 12 participants (Guest et al., 2006). Our sample was female-biased, whereas there is approximate gender balance in IBD presentation in the general population (Kappelman et al., 2007). The participants also had very disparate ages and lengths of time with the condition, which while helpful to gain an understanding of experiences across the population, may limit transferability of findings to more specific groups.

Additionally, participants were self-selected; as volunteers, they were potentially more open and insightful about their emotional responses to their condition. Other potential respondents may have anticipated disgust responses from healthcare staff and decided against participation. These individuals may be those experiencing greater self-consciousness, so this is a potential source of bias in the sample (Reynolds et al., 2015). It is also important to consider that participants will likely vary in their ability to identify and express experienced emotions, and in the way that they experience them, which will have shaped the themes developed in this analysis.

## Conclusions

The present thematic analysis investigated the experience of self-conscious emotions in people living with IBD. Participants experienced threats to their identity through lack of control due to effects of IBD, and through other people’s infantilising and diminishing behaviours, which discouraged help-seeking. Participants described feelings of shame when the disease was exposing, humiliation and anger when others questioned them, and guilt when their IBD affected relationships. These findings may be important in shaping psychological and therapeutic support for people with IBD; for example, compassion-focussed therapies may be particularly helpful, and should be the focus of successor research.
